# TMPRSS2, a novel host-directed drug target against SARS-CoV-2

**DOI:** 10.1038/s41392-022-01084-x

**Published:** 2022-07-23

**Authors:** Christian Keller, Eva Böttcher-Friebertshäuser, Michael Lohoff

**Affiliations:** 1 Institute of Virology, Philipps University Marburg, Marburg, Germany; 2 Institute of Medical Microbiology and Hygiene, Philipps University Marburg, Marburg, Germany

**Keywords:** Drug development, Target identification

In a recent study in *Nature*,^1^ Shapira et al. reported that the peptidomimetic compound N-0385 inhibited SARS-CoV-2 infection in vitro in remarkably low concentrations by blocking the host cell protease TMPRSS2, which mediates proteolytic cleavage of the SARS-CoV-2 major surface glycoprotein spike (S). Accordingly, N-0385 protected experimentally infected mice from severe SARS-CoV-2 pathology.^1^

The combat against COVID-19 still largely relies on vaccination against SARS-CoV-2. Yet, some people cannot be vaccinated or mount low or no responses against the vaccine, e.g., elderly people above the age of 80.^2^ Antigenic escape by, e.g., the Omicron variant (B.1.1.529) may jeopardize passive immunization by monoclonal antibodies, calling for complementary concepts in prophylaxis and therapy. So far, these have concentrated on agents that directly interfere with viral replication, e.g., by inhibition of the viral 3CL^pro^ protease (paxlovid), by inhibition of the RNA-dependent RNA polymerase (remdesivir), or by introducing copying errors (molnupiravir).

**Fig. 1:**
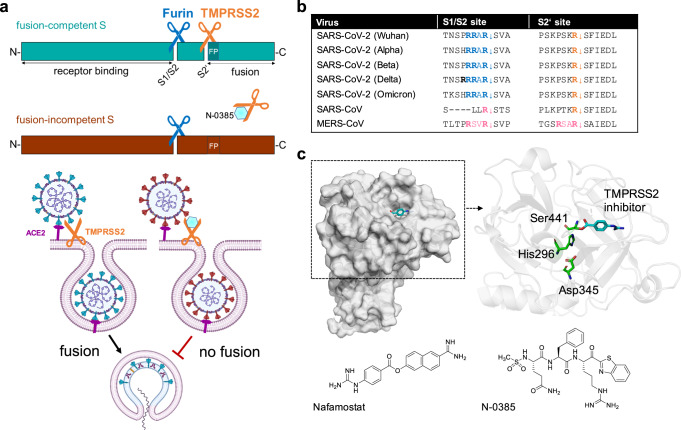
Inhibition of SARS-CoV-2 spike cleavage by TMPRSS2 inhibits virus entry. **a** Upper panel: Schematic representation of SARS-CoV-2 S cleavage by furin at the S1/S2 site and subsequent cleavage at the S2’ site by TMPRSS2. Cleavage at the S2’ site exposes the fusion peptide (FP), priming S for membrane fusion (cyan-colored S). Inhibition of TMPRSS2 by N-0385 (blue hexagon) causes incomplete cleavage of S (red-colored S). Lower panel: Complete cleavage of S supports membrane fusion and the release of the viral genome into the target cell. TMPRSS2 inhibition results in incomplete S cleavage and thus prevents fusion and virus entry. **b** Alignment of the amino acid sequences at the S1/S2 and S2‘ sites of different SARS-CoV-2 variants of concern, zoonotic SARS-CoV and MERS-CoV. Amino acid motifs highlighted in blue and orange are cleaved by furin and TMPRSS2, respectively. Motifs highlighted in pink are most likely cleaved by TMPRSS2. **c** Crystal structure of human TMPRSS2 (SRCR and serine protease domain) in complex with nafamostat (cyan, PDB: 7MEQ; upper left panel). Catalytic domain (cartoon style) of TMPRSS2 (upper right panel). The catalytic triad (green stick model) and nafamostat (cyan) are indicated. Structures of nafamostat (PubChem CID 4413) and N-0385 (PubChem CID 135169285, lower panels)

Shapira et al. have now demonstrated that it is possible to therapeutically exploit a host cell process that is pivotal in SARS-CoV-2 infection.^1^ Viral infection not only requires binding of S to its receptor angiotensin-converting enzyme 2 (ACE2) but also cleavage of S at two distinct sites by host cell proteases: First, furin (and related pro-protein convertases) cleaves at a polybasic site, generating the S1 and S2 subunits, which remain non-covalently linked (S1/S2 site, Fig. 1a). Upon receptor binding, another cleavage site (S2’ site), located immediately upstream of the hydrophobic fusion peptide, is exposed, and a second cleavage is mediated by TMPRSS2 and related proteases (Fig. 1a). This enables exposure of the fusion machinery located in the S2 subunit.^3^ Previous work by Bestle et al. had indicated that synergistic inhibition of furin by the peptidomimetic inhibitor MI-1851 and TMPRSS2 by MI-432 or MI-1900 in micromolar concentrations significantly reduced viral replication in vitro.^3^ Shapira et al. now added to this work by demonstrating that inhibition of TMPRSS2 by peptidomimetic tetrapeptide compounds with ketobenzothiazole warheads is effective against SARS-CoV-2 even in nanomolar concentrations.^1^ Mechanistically, TMPRSS2 inhibition results in incompletely cleaved, fusion-incompetent SARS-CoV-2 viruses that are unable to infect new host cells (Fig. 1a).

From eight candidates, the authors identified N-0385 as the most potent inhibitor of TMPRSS2 and viral infection in vitro. Its antiviral action was verified in human intestinal biopsy-derived colonoids, a recently developed primary cell system. With a selectivity index of >10^6^, inhibition was highly specific for TMPRSS2. Importantly, the authors demonstrated the antiviral activity of N-0385 against four variants of concern (Alpha, Beta, P1, and Delta).

For in vivo studies, the authors continued in a humanized K18-hACE2 mouse model, in which transgenic expression of human ACE2 is controlled by the cytokeratin 18 promoter. Intranasal administration of N-0385 before and during SARS-CoV-2 infection significantly reduced mortality and morbidity.^1^ Also, a shorter prophylactic treatment with only four administrations (7.2 mg/kg) and initiated before infection, reduced viral titers in the lungs and protected all treated mice from a lethal outcome. Interestingly, even a single treatment with N-0385 (14.4 mg/kg) given 12 h post infection with the Delta variant reduced weight loss and viral titers in the lungs of infected mice.

The data presented by Shapira et al. suggest spike maturation by inhibiting TMPRSS2 as a potent drug target in COVID-19 when the inhibitor is given before or early in infection. Most currently licensed antivirals are applicable in the early phase, until day 5–7 after the onset of symptoms. This requirement to treat early was thus not overcome by the present study, and the therapeutic window of TMPRSS2 inhibition needs to be studied more extensively. A prophylactic application, however, emerges as a realistic scenario. As an improvement to previously described TMPRSS2 inhibitors that required micromolar concentrations, N-0385 acted against SARS-CoV-2 in the nanomolar range and with increased specificity.

The study also revealed some heterogeneity between male and female groups; females appeared either less susceptible to infection or more susceptible to treatment.^1^ Future studies should therefore address sex differences in antiviral therapy. Synergistic treatments with directly acting antivirals or other protease inhibitors (e.g., aprotinin) will equally be a worthwhile field of study.

As an advantage over virus-directed therapies, host-directed targets have a low potential for resistance: The TMPRSS2 cleavage site of the SARS-CoV-2 spike protein has remained very conserved over the pandemic (Fig. 1b), suggesting that N-0385 should retain a high potency against future variants of concern. Another advantage to target TMPRSS2 is its activating function in other viral infections (e.g., other coronaviruses, influenza virus), which expands the field of potential applications for TMPRSS2 inhibitors. The recently described crystal structure of TMPRSS2 might facilitate further improvements by structure-based drug design (Fig. 1c).

Broad-spectrum serine protease inhibitors against COVID-19 that were recently evaluated in clinical trials are intravenous nafamostat, a synthetic inhibitor, and aerosolized aprotinin, a protease inhibitor from bovine lung. For nafamostat, one study demonstrated a small effect with respect to recovery rate and mortality in high-risk COVID-19 patients requiring oxygen treatment.^4^ Adverse effects, which may restrict its use as an intravenous drug, included catheter site phlebitis, hyponatremia, and respiratory failure.^4^ More encouraging data came from the aprotinin study: aerosolized aprotinin reduced the length of hospital admission and oxygen requirement.^5^ Importantly, no adverse reactions were observed. Thus, inhalation could be a safer way to administer protease inhibitors against COVID-19, tailored to reach TMPRSS2 that is expressed on the cell surface of target cells in the airways. While the physiological role of TMPRSS2 in humans is not clear, it is non-essential in mice. Since redundant proteases should take over its function, inhibition with selective compounds is likely not to produce relevant side effects.

Taken together, Shapira et al. have developed a potent TMPRSS2 inhibitor and provided robust evidence that host-directed inhibition of TMPRSS2 by the peptidomimetic compound N-0385, thus preventing spike maturation, is effective against SARS-CoV-2 in vitro and in vivo, including variants of concern. People without vaccination, with poor vaccine responses or with an increased risk for severe infection courses are likely to benefit most from TMPRSS2 inhibition, which at this stage ought to be initiated soon after exposure.
